# Dupilumab-induced psoriasis in a patient with atopic dermatitis successfully treated with Upadacitinib: A case report

**DOI:** 10.1177/2050313X251317811

**Published:** 2025-02-03

**Authors:** Katrina D Cirone, Fiona E Lovegrove

**Affiliations:** 1 Schulich School of Medicine & Dentistry, Western University, London, ON, Canada; 2 Lovegrove Dermatology, London, ON, Canada

**Keywords:** Atopic dermatitis, psoriasis, dupilumab, upadacitinib, drug reaction

## Abstract

Dupilumab, a monoclonal antibody that targets interleukin-4 and interleukin-13, is one of the approved biologic treatments for moderate-to-severe atopic dermatitis. While it is extremely well tolerated with low rates of adverse events, there have been reports of patients with atopic dermatitis managed on Dupilumab developing new-onset psoriasis. The development of psoriasis in patients with atopic dermatitis on Dupilumab therapy is believed to occur due to a decrease in T helper 2 activity and resultant dysregulation of the T helper 1 and T helper 17 pathways involved in psoriasis pathogenesis. Upadacitinib, an oral, selective Janus kinase 1 inhibitor, approved for use in moderate-to-severe atopic dermatitis as well as psoriatic arthritis may have a potential role in psoriasis treatment. We describe a case of a patient who initially presented with longstanding atopic dermatitis that underwent a psoriasiform switch while managed on Dupilumab and is currently stable on Upadacitinib.

## Introduction

Atopic dermatitis (AD) is a chronic, inflammatory skin condition characterized by skin barrier dysfunction and an underlying perpetuation of cytokine production and T helper 2 (Th2)-driven autoimmune and inflammatory processes.^1,2^ Dupilumab, a monoclonal antibody targeting interleukin (IL)-4 and IL-13, is one of the approved biologic treatments for moderate-to-severe AD.^3,4^ Although it is an effective and well-tolerated treatment for AD with low rates of adverse events, in recent years there have been reports of patients with AD managed on Dupilumab developing new-onset psoriasis.^5,6^ Development of psoriasis in patients with AD on Dupilumab therapy is believed to occur due to a blockade of the Th2 inflammatory cascade and resultant upregulation of the Th1/Th17 pathway, which is involved in psoriasis pathogenesis.^5,7^

Upadacitinib, an oral, selective Janus kinase 1 (JAK1) inhibitor is approved for use in moderate-to-severe AD as well as other immune-mediated inflammatory conditions, including psoriatic arthritis, ulcerative colitis, and Crohn’s disease.^8–10^ JAK inhibitors have broader immunomodulatory properties than Dupilumab and have been found to successfully treat several patients with clinical overlap between AD and psoriasis.^11^

We describe a case of a patient with longstanding AD and allergic rhinitis who presented after approximately 2 years of almost-clear skin on Dupilumab treatment with a new onset of widespread psoriasiform plaques, confirmed as psoriasis on histopathology. Following this new clinical presentation, Dupilumab was discontinued and she was treated with topical therapy and oral Apremilast without benefit. Subsequently, she initiated treatment with Upadacitinib with excellent response and has had clear skin with no adverse effects for over a year.

## Case report

A 66-year-old female presented in October 2016 with a longstanding history of widespread AD involving her scalp, face, trunk, and extremities, previously inadequately managed on betamethasone ointment and hydroxyzine. The ; BSA: Body Surface Area remainder of her medical history was negative apart from seasonal allergies, and she was not on any additional medications. She reported no family history of dermatitis or related autoimmune conditions. Her review of systems was unremarkable. A clinical diagnosis of AD with lichenification and prurigo nodules was made at the time of presentation and later confirmed by biopsy as lichenified dermatitis in November 2016.

Initial treatments between October 2016 and May 2019 consisted of various topical corticosteroids (betamethasone valerate, clobetasol propionate cream and spray, desonide, and fluocinonide), tacrolimus, crisaborole, and oral antihistamines (hydroxyzine and bilastine) (Table 1). Her disease course was complicated by multiple *Staphylococcus aureus* superinfections which were all acutely managed with oral cefalexin and fusidic acid for long-term infection prevention. In April of 2019, she returned to the clinic with a flare of extensive dermatitis (body surface area (BSA) 60%) and received a 4-week tapering course of oral prednisone 20 mg. In May 2019, she continued to demonstrate poor disease control and subsequently initiated treatment with Dupilumab 300 mg every 2 weeks. Initially, she reported an excellent response (BSA 0%, Eczema Area and Severity Index (EASI) 0) and experienced no adverse effects during the first 2 years of treatment.

**Table 1.** Summary of treatments, dosage, and response during the case report.

Date	Treatment and dosage	Response
October 2016	Topical (betamethasone valerate, clobetasol propionate cream and spray, desonide, fluocinonide, tacrolimus, crisaborole)	Minimal response
April 2019	Prednisone 20 mg OD for a 4-week taper	Minimal initial response, no significant improvement
May 2019	Dupilumab 300 mg SC every 2 weeks	Excellent initial response over the first 2 years, not sustained
September 2021	Apremilast 30 mg po BID and topicals (calcipotriol, betamethasone dipropionate, and hydrocortisone)	Good initial response, not sustained
June 2022	Upadacitinib 15 mg po OD, ongoing	Excellent response, currently sustained

EASI: Eczema Area and Severity Index; PASI: Psoriasis Area Severity Index; BSA: Body Surface Area.

Response was determined using a combination of severity scores (EASI, PASI, BSA) and descriptions listed in the patient chart.

**Figure 1.**
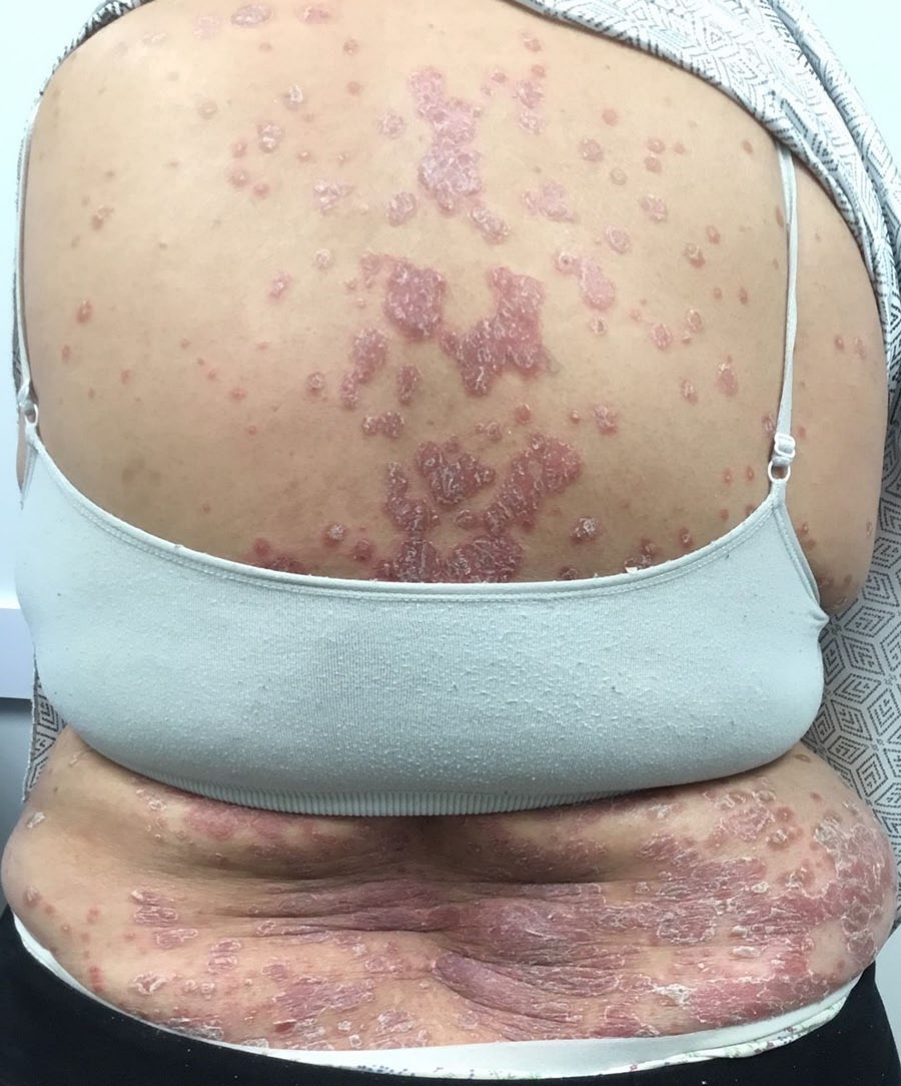
Clinical image of a widespread psoriasiform eruption characterized by well-demarcated erythematous plaques with overlying silvery scale on the patient’s back.

She later presented to the clinic in March 2021 with new-onset, widespread, and well-demarcated erythematous plaques with a silvery scale, resembling plaque psoriasis (BSA 55%, Psoriasis Area Severity Index 11.6) (Figure 1). She had no personal history of psoriasis and had no known family members with the condition. A lesional biopsy was obtained which confirmed the diagnosis of psoriasis on histopathology. She discontinued Dupilumab and began treatment with Apremilast 30 mg twice a day as well as topical calcipotriol, betamethasone dipropionate, and hydrocortisone without benefit and after a year of treatment, her skin began to resemble AD more closely than psoriasis, as seen clinically by the presence of dermatitic plaques and lichenification located on her face, neck, and upper back (BSA 10%, EASI 9.4). Initially, she had good control of Apremilast with clear skin, however; in June of 2022, she returned to the clinic as she had developed dermatitis plaques. Subsequently, she discontinued Apremilast and began treatment with Upadacitinib 15 mg once daily. Since the initiation of treatment with Upadacitinib over a year ago, she continues to demonstrate excellent disease control (BSA 0%, EASI 0) and no adverse events.

## Discussion

We describe a case of new-onset psoriasis that developed in the setting of Dupilumab treatment of AD, successfully treated with Upadacitinib. While both psoriasis and AD are common T cell-mediated inflammatory conditions, the immune imbalance in these diseases is thought to exist at opposite ends of the Th1 and Th2 spectrum where psoriasis primarily features Th1/Th17 cells while AD is a mainly Th2-mediated disease, with more Th1 involvement seen in chronic cases.^7,12^ Dupilumab inhibits Th2-mediated inflammation, which in our patient’s case, may have driven an immunologic shift toward the Th1/Th17 pathway involved in psoriasis pathogenesis and the resultant clinical presentation of psoriasis.^13^ It is currently hypothesized that inhibition of Th2 with Dupilumab treatment causes the Th1 response to become more pronounced.^14,15^ Although no clear explanation exists for the association between Dupilumab’s Th2 inhibition and the implication on psoriasis pathogenesis, prior case reports demonstrating Dupilumab-induced psoriasis in patients with AD also propose a possible shift from a Th2 to Th1-mediated inflammatory response.^14,15^

Upadacitinib, an oral selective JAK1 inhibitor has been approved for use in moderate-to-severe AD and psoriatic arthritis.^8,9^ Upadacitinib’s target of inhibition—JAK1—is known to be involved in signaling through interferon-alpha/beta, IL-6, IL-10, and IL-22 receptors, and therefore, Upadacitinib has broader immunomodulatory properties than Dupilumab.^11^ In the multicenter randomized placebo-controlled phase 3 trial (Heads UP), Upadacitinib demonstrated superior efficacy to Dupilumab in patients with moderate-to-severe AD as indicated by achievement of EASI75, EASI100, and improvement in worst pruritus numerical rating scale with no new adverse events.^8^ Further, a recent real-world multicenter cohort study found patients with AD who had previously failed systemic therapies including Dupilumab had a significant improvement in disease severity as indicated by EASI75, EASI90, and EASI100 when treated with Upadacitinib.^16^

To date, no clinical trials investigating the role of Upadacitinib in the management of cutaneous psoriasis have been completed. A case series of concomitant psoriasis and AD, successfully treated with Upadacitinib, demonstrated that JAK1 inhibition was of benefit in cases with clinical overlap between these two conditions, with all patients achieving complete remission of both diseases with no adverse events seen after at least 32 weeks of follow-up.^10^

The uncommon occurrence of AD to psoriasis “switches” on Dupilumab therapy is relatively well described; however, the pathogenesis and management are not. A previous case report and literature review of patients who developed psoriasis in the setting of Dupilumab therapy indicated that the majority of patients benefited from topical monotherapy.^17^ In the case we presented here, the patient did not respond adequately to topical therapy, and systemic treatment was warranted. The dilemma was whether to target therapy toward the psoriasis or AD pathway and ultimately, use of Upadacitinib was of benefit. This case contributes to the current knowledge base for JAK inhibitors and suggests that patients who develop psoriasis on IL-4/IL-13 biologic therapy for AD can be treated safely and effectively with JAK inhibitors. Further, longitudinal studies are needed to further characterize the role of Upadacitinib in the management of psoriasis.
